# Pervasive Rise of Small-scale Deforestation in Amazonia

**DOI:** 10.1038/s41598-018-19358-2

**Published:** 2018-01-25

**Authors:** Michelle Kalamandeen, Emanuel Gloor, Edward Mitchard, Duncan Quincey, Guy Ziv, Dominick Spracklen, Benedict Spracklen, Marcos Adami, Luiz E. O. C. Aragão, David Galbraith

**Affiliations:** 1 School of Geography, University of Leeds, Leeds, LS2 9JT UK; 2 School of GeoSciences, University of Edinburgh, Edinburgh, EH9 3FF UK; 3 School of Earth and Environment, University of Leeds, Leeds, LS2 9JT UK; 4 Instituto Nacional de Pesquisas Espaciais (INPE), Belém, Pará CEP: 66077-830 Brazil; 5 Instituto Nacional de Pesquisas Espaciais (INPE), Sao José dos Campos, São Paulo, CEP: 1227-010 Brazil; 6 College of Life and Environmental Sciences, University of Exeter, Exeter, EX4 4RJ UK

## Abstract

Understanding forest loss patterns in Amazonia, the Earth’s largest rainforest region, is critical for effective forest conservation and management. Following the most detailed analysis to date, spanning the entire Amazon and extending over a 14-year period (2001–2014), we reveal significant shifts in deforestation dynamics of Amazonian forests. Firstly, hotspots of Amazonian forest loss are moving away from the southern Brazilian Amazon to Peru and Bolivia. Secondly, while the number of new large forest clearings (>50 ha) has declined significantly over time (46%), the number of new small clearings (<1 ha) increased by 34% between 2001–2007 and 2008–2014. Thirdly, we find that small-scale low-density forest loss expanded markedly in geographical extent during 2008–2014. This shift presents an important and alarming new challenge for forest conservation, despite reductions in overall deforestation rates.

## Introduction

Amazon rainforest deforestation has been a major issue in the environmental agenda, driven by concerns about deteriorating ecosystem services, biodiversity loss and increasing carbon emissions^1–3^. For example, loss of forest cover has been shown to result in sharp reductions in species richness in southern Amazonia^4^. Furthermore, as the Amazon Basin accounts for ~5.4 million km^2^ of contiguous tropical forests^5^ and 150–200 Pg of carbon^6^, changes in land use in Amazonia are expected to have significant regional and global climatic consequences^7,8^, with potential impacts on climate and agriculture in other parts of South America and, via teleconnections, in other continents^9^.

Despite its significance, comprehensive assessments of the dynamics of forest loss across the entire Amazon region are rare. In fact, the majority of regional-level studies to date have focused only on the Brazilian Amazon^1,10–13^. In part, this is due to the highly successful PRODES (Monitoramento do Desmatamento na Amazônia Legal por Satélite) programme, operated by the Brazilian Institute for Space Research (INPE), which provides annual estimates of deforestation for the entire Brazilian Amazon since 1988 based on 30m-resolution Landsat satellite images^14^. The PRODES data have been used to highlight the marked decline in deforestation in the Brazilian Amazon over the last decade, where deforestation was reported to have fallen from a record 27,772 km^2^ in 2004 to 4,571 km^2^ in 2012^14^. In this regard, PRODES enabled government actions through the Plano de Prevenção e Controle do Desmatamento na Amazônia (PPCDAm) programme that led to a revolutionary impact on reducing deforestation in the Brazilian Amazon^15^. This steered renewed optimism about Brazil’s capacity to contain Amazonian deforestation, with some authors predicting an end to deforestation of the Brazilian Amazon by 2020^13^.

PRODES-based studies, however, do not provide a complete picture of deforestation dynamics within the Basin for several reasons: 1) they do not consider deforestation events <6.25 ha; thus, smaller disturbances such as those associated with subsistence agriculture and artisanal or small-scale mining (typically <5 ha) are not presented by PRODES unless accumulated over several years to >6.25ha^16^, 2) they only consider primary forests and do not account for secondary or regenerating forests and 3) they are restricted to Brazil^17^.

Furthermore, trends in Brazil may not reflect those occurring in other Amazonian countries or regions. In Bolivia, for example, forest loss has accelerated from ~400 km^2^ y^−1^ in the 1960s to 2900 km^2^ y^−1^ by 2004^18^; while deforestation in the Colombian Amazon has also been reported to increase over the last decade^19,20^. Other studies have highlighted sub-national, regional increases in deforestation. For example, significant increases in deforestation have been documented in the Madre de Dios region of Peru associated with a rapid surge in small-scale gold mining^21^.

Recent advances in data availability and processing power have enabled the emergence of high-resolution regional and global forest cover change datasets spanning over ten years of data, such as the University of Maryland’s Global Forest Change (GFC) product^22^, based on hundreds of thousands of 30m-resolution Landsat satellite scenes, allowing for in-depth assessment of forest loss extent and temporal dynamics. Here, we make use of the GFC product to comprehensively evaluate changes in the spatial patterns of forest loss across all of Amazonia over a 14-year period (2001–2014). We specifically consider temporal and spatial shifts in 1) forest loss hotspots (clusters of high rates of deforestation), 2) size of forest loss patches and 3) the geographical density of forest loss events. These analyses provide critical insights into shifts in potential drivers of deforestation^11^ and establish a barometer for evaluating the effectiveness of conservation strategies such as protected areas.

## Results

### Spatio-temporal evolution of forest loss hotspots

**Figure 1:**
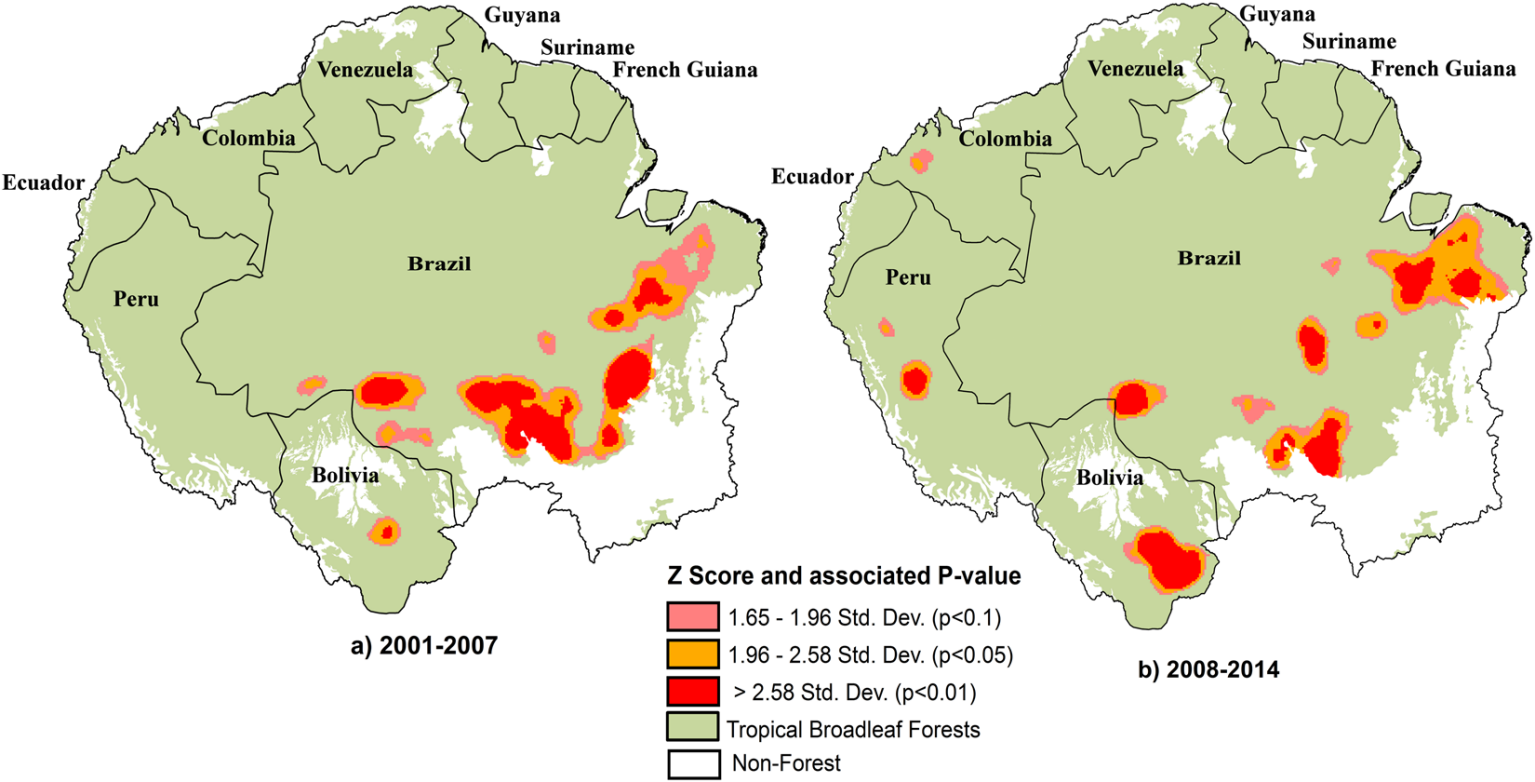
Hotspots of Amazonian forest loss based on Getis Ord Gi* z-scores for GFC data for two time periods: 2001–2007 and 2008–2014 using ArcGIS 10.4.1 (www.esri.com). Higher values indicate increased clustering of deforestation patches.

Our analysis reveals important shifts in statistically significant forest loss hotspots (areas with concentrated forest loss activity) across Amazonia using a local clustering statistic, the Getis-Ord Gi* metric (see Methods). This metric has been utilized previously for hotspot analysis mainly in the health and urban sectors^23,24^. Over the first half of our study period (2001–2007), spatial hotspots of deforestation were concentrated along the widely known ‘arc of deforestation’ extending across the southern rim of the Brazilian Amazon from Pará to Rondônia, with an especially large hotspot in Mato Grosso (Fig. 1). The only hotspot of note outside of Brazil during the 2001–2007 period was a relatively small region in western Santa Cruz state and bordering Beni state in Bolivia.

The distribution of deforestation hotspots during the 2008–2014 period differs from that of the 2001–2007 period due to three major notable features: 1) the weakening of the Brazilian ‘arc of deforestation’ as a deforestation hotspot, 2) the southward expansion of the Bolivian hotspot of deforestation and 3) the emergence of a new deforestation hotspot in Amazonian Peru. The apparent disappearance of the traditional arc of deforestation is driven by a marked decline in the importance of Mato Grosso, and to a lesser extent Pará, as a forest loss hotspot. The Bolivian hotspot expanded rapidly from covering an area of ~300 km^2^ in 2001-2007 to an area of ~9560 km^2^ in 2008-2014, thus representing the largest deforestation hotspot (at 99% confidence levels) over the Amazon during that period (Fig. 1, SI Figure S1). The Peruvian forest loss hotspot is much smaller in area (2066 km^2^) than the Bolivian hotspot and only emerged during the second half of the study period. Accompanying the Peruvian hotspot is the emergence of a smaller, statistically weaker hotspot in western Colombia, also not evident during the first half of the study period.

### Temporal patterns of forest loss patch size

We also found that mean forest loss patch size declined across Amazonia over our study period. Between 2001 and 2014, the mean forest loss patch size across the study region was 10.25 ha but varied considerably across countries, ranging from 0.5 ha in Ecuador to 15.6 ha in Brazil, (SI Figure S2). Bolivia was the only country besides Brazil where mean forest loss patch size was >1 ha. We found that a majority (96.4%) of deforested patches were below the 6.25 ha threshold considered by PRODES, with a large number of these (81.1%) below 1 ha. In area terms, patches below 6.25 ha accounted for ~39% and ~34% of total Amazonian and Brazilian Amazon forest loss across our study period respectively.

Mean patch size trajectories across time in Brazil and Bolivia were similar, increasing from 2001 up to 2004 and declining thereafter (SI Figure 2). In both countries, mean patch size was significantly greater in 2001–2007 than in 2008–2014 (W = 49, *p* = 0.0005). Significant declines in mean size of forest loss patches between 2001–2007 and 2008–2014 were also found for Venezuela (W = 41, *p* = 0.038), French Guiana (W = 45, *p* = 0.006) and Guyana (W = 43, *p* = 0.017), but the total deforested area in these countries was much lower than in Brazil and Bolivia.

**Figure 2:**
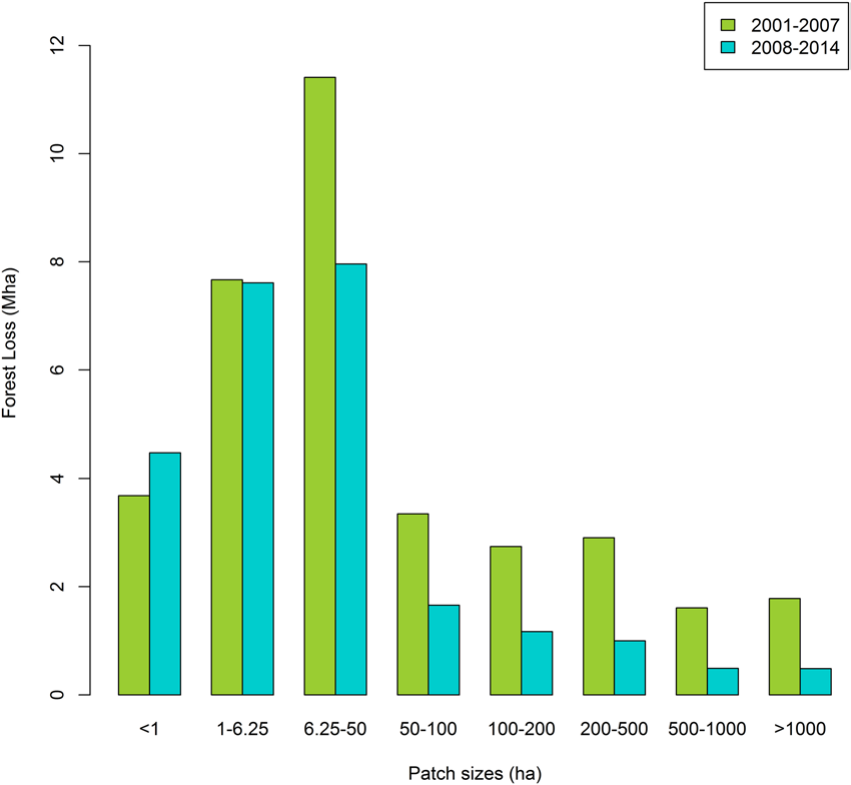
Change in deforested area (ha) of different size categories between 2001–2007 and 2008–2014 across Amazonia using the GFC dataset.

The declining mean patch size of forest loss across Amazonia reflects both a decline in the number of larger forest loss patches and an increase in the number of smaller patches (Fig. 2). The number of very large (>500 ha) deforested patches declined significantly over time (W = 49, *p* > 0.001) by 67% between 2001–2007 and 2008–2014 (Table 1). This is driven by a 72% decline in very large forest loss patches in Brazil, the country with by far the greatest total number of forest loss patches, between the two time periods. Similarly, the number of very large forest loss patches in Bolivia also declined by 25% between both of our study periods.

**Table 1:** Changes in the number of patches between 2001–2007 and 2008–2014 using the *Hansen et al*. GFC product. The significance of the difference between the two time periods (2001–2007 and 2008–2014) were estimated using the Wilcoxon signed rank test.

**Patch Size**	**Mean number of forest loss patches**		**% Difference between time periods**	**Statistical Significance**
	**2001**–**2007**	**2008**–**2014**		
<1 ha	2069303	2709837	30.95	W = 13, *p* = 0.16
1–6.25 ha	445736	457348	2.61	W = 23, *p* = 0.9
6.25–50 ha	110007	82481	−25.02	W = 44, *p* = 0.01
50–100 ha	6965	3487	−49.93	W = 49, *p* < 0.001
100–200 ha	2870	1230	−57.12	W = 49, *p* < 0.001
200–500 ha	1388	482	−65.31	W = 49, *p* < 0.001
500–1000 ha	336	102	−69.71	W = 47, *p* = 0.002
>1000 ha	146	39	−73.24	W = 46, *p* = 0.004

There was also a significant decline in forest loss patches of large and intermediate size (50-500 ha) by 27% (W = 44, *p* = 0.01), while the number of small forest loss patches (<6.25 ha) increased by ~34% (W = 13, *p* = 0.16) between our two study periods (Table 1). The overall pattern of increasing number of small forest loss patches was seen across all Amazonian countries (SI Figure S3), although the multi-annual patterns of change varied according to country.

In Brazil and Bolivia, the number of small forest loss patches increased gradually throughout the study period while in northern Amazonian countries (French Guiana, Guyana, Suriname, Venezuela) and in western Amazonian countries (Colombia, Ecuador, Peru) there were pronounced increases in small forest loss events in 2012, with forest loss rates rebounding back to close to previous levels in 2013. For example, the number of forest loss patches <1 ha increased by 354% in French Guiana and 318% in Suriname between 2011 and 2012.

### Geographical spread of deforestation events

**Figure 3:**
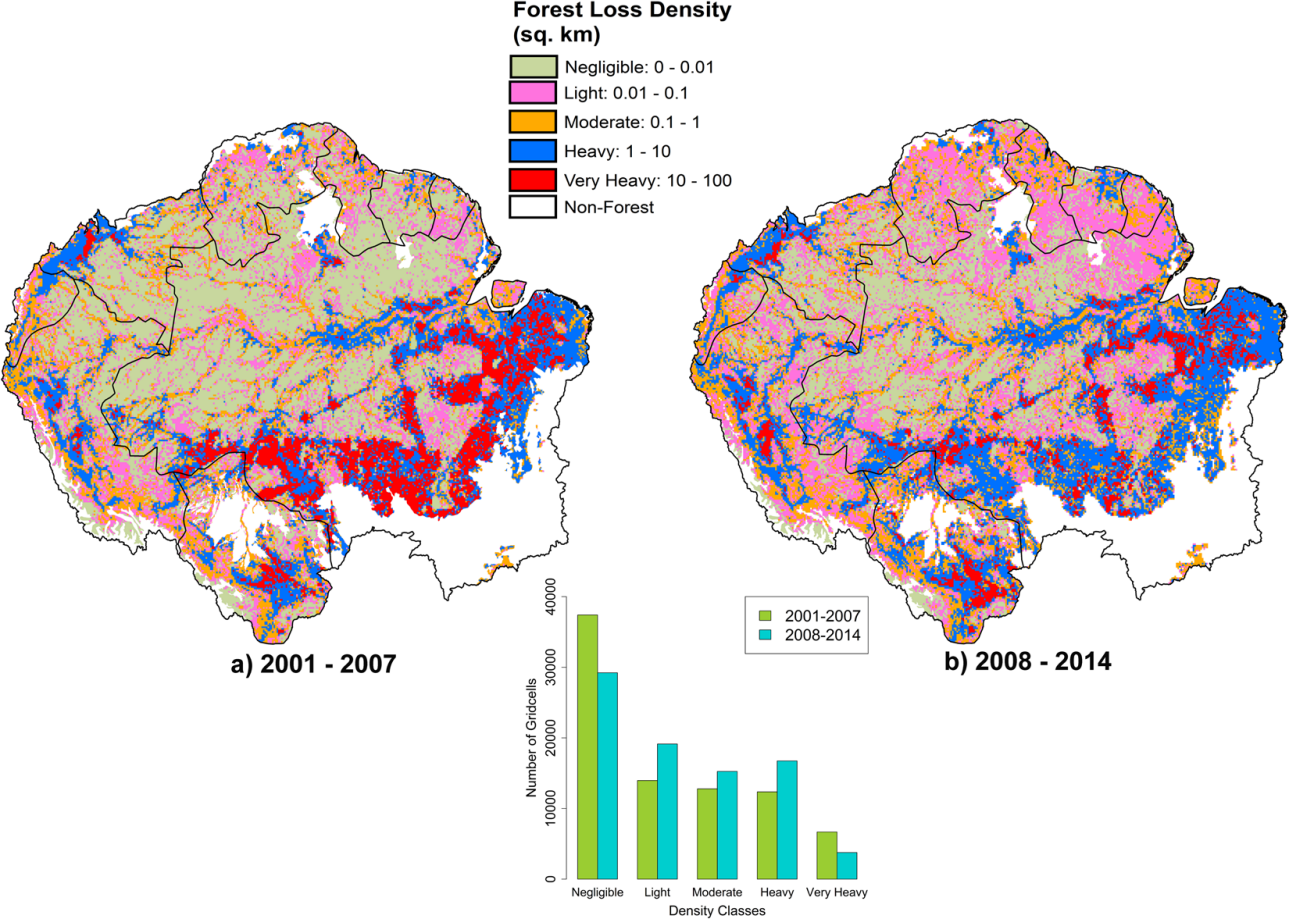
Forest loss density (km^2^ forest loss per 100 km^2^ land area) in Amazonia, as calculated using the GFC product for two time periods: (**a**) 2001–2007 and (**b**) 2008–2014 using ArcGIS 10.4.1 (www.esri.com). Histogram indicates the number of gridcells for each density class.

Considerable changes were also observed in the geographical patterns of deforestation density between our two study periods (Figs 3, SI Figures 4 and 5). Whereas in 2001–2007, 45% of the 10 × 10 km gridcells in our study region were categorised as having negligible forest loss (<0.01 km^2^ per 100 km^2^), this declined to 35% in 2008–2014, despite overall Basin-scale declines in deforestation. The proportion of gridcells experiencing ‘very heavy’ deforestation (>10 km^2^ per 100 km^2^) declined by 66.7%, from 9% in 2001–2007 to 4.5% in 2008–2014. Conversely, an increase in the number of gridcells experiencing ‘light’ (0.01–0.1 km^2^ per 100 km^2^) and ‘moderate’ deforestation (0.1–1 km^2^ per 100 km^2^) was observed, increasing from 19% and 17.5% of gridcells in 2001–2007 to 23% and 18% in 2008–2014 respectively. The number of gridcells classified as having ‘heavy’ (1–10 km^2^ per 100 km^2^) deforestation increased from 17% to 20% of gridcells between the two time periods.

**Figure 4:**
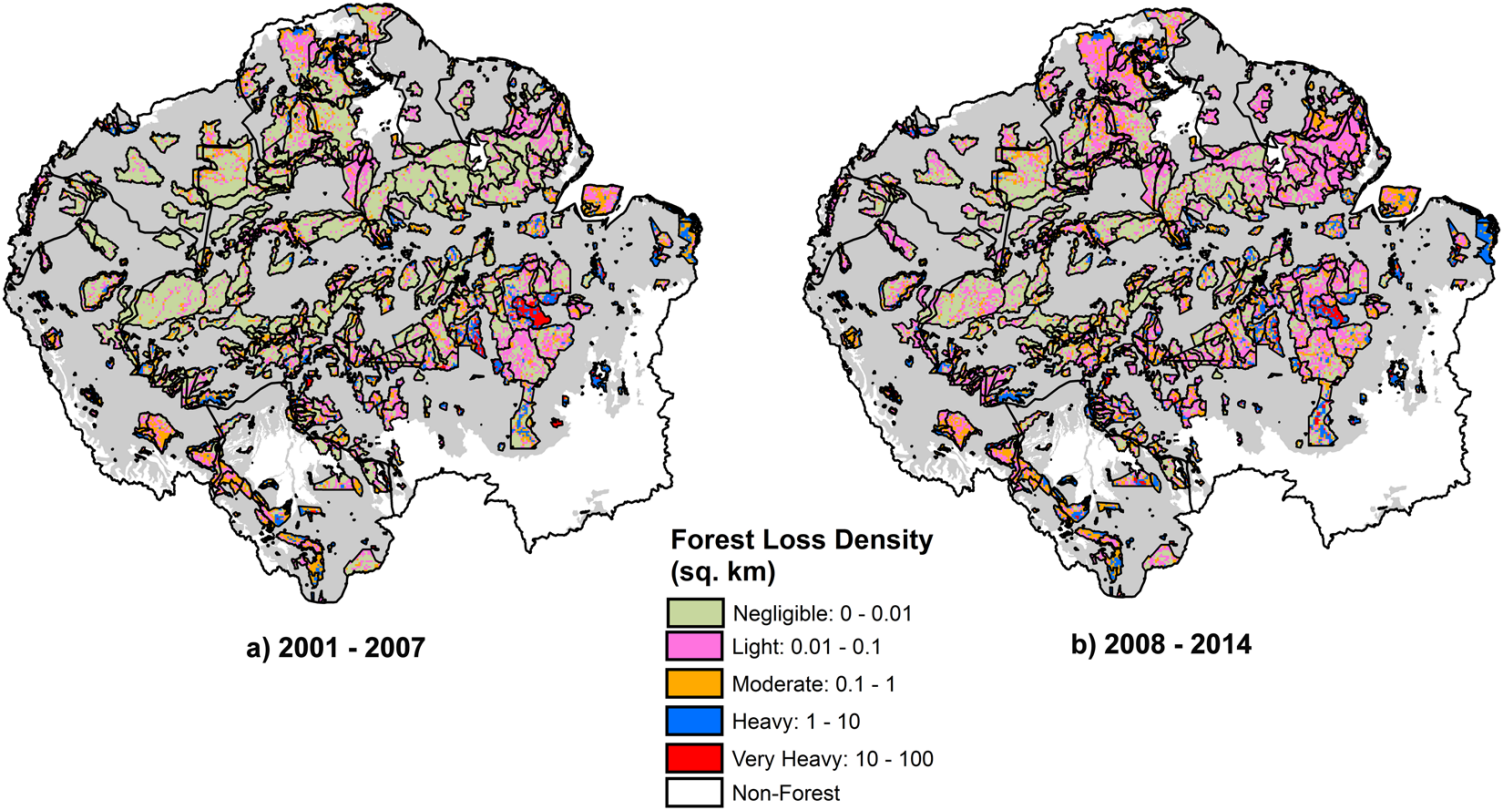
Forest loss density (km^2^ forest loss per 100 km^2^ land area) in Protected Areas across Amazonia, as calculated using the GFC product for two time periods: (**a**) 2001–2007 and (**b**) 2008–2014 using ArcGIS 10.4.1 (www.esri.com). For visualisation purposes, deforestation outside of protected areas is not shown. [Source of PA shapefiles: UNEP-WCMC and IUCN (2016), Protected Planet: The World Database on Protected Areas (WDPA), October, 2016, Cambridge, UK: UNEP-WCMC and IUCN. Available at: www.protectedplanet.net].

We tested whether patterns observed in protected areas differed from patterns observed outside of protected areas but found that temporal patterns were similar inside of protected areas to those outside (Fig. 4). Inside protected areas across the Amazon, the number of gridcells with negligible forest loss fell by 10% between 2001–2007 and 2008–2014 (W = 13, *p* = 0.2) while ‘light’ and ‘moderate’ deforestation increased by 18.4% (W = 4, *p* = 0.3) and 30% (W = 0, *p* = 0.02) respectively. Conversely, ‘very heavy’ deforestation declined by 54% in protected areas between our two study periods (W = 12, *p* = 0.3), in line with patterns outside of protected areas.

Overall, our results suggest a progressive encroachment of low-density, small-scale deforestation into areas of Amazonia such as Amazonas, Roraima and Amapá (Fig. 3, SI Figure S5), which historically have had negligible deforestation, as well as the northern Amazonian countries of French Guiana, Guyana and Suriname.

### Large-scale deforestation temporal patterns

Overall, Amazonian forest loss based on GFC declined between our two study periods (SI Figure S6), from 238 km^2^ yr^−1^ in 2001–2007 to 177 km^2^ yr^−1^ in 2008–2014 (W = 43, *p* = 0.017). This is driven by statistically significant reductions in forest loss in Brazilian Amazonia which was 49.5% lower in 2008–2014 than 2001–2007 (W = 48, *p* = 0.001) but is offset somewhat by increasing forest loss in the non-Brazilian Amazon, which was 35% higher in 2008–2014 than 2001–2007 (W = 1, *p* = 0.001).

During the first half of the study period (2001–2007), we found that forest loss outside of Brazil accounted for 9.7% of total Amazonian forest loss, rising to 13.8% during the second half of the study period (2008–2014). The largest increases in forest loss occurred in Peru (106.73 km^2^ yr^−1^) and Bolivia (66.68 km^2^ yr^−1^), which together accounted for 72.3% of the increasing deforestation trend in the non-Brazilian Amazon (SI Table S1). Although we observed significant differences between our focal study periods, we note that there is also evidence of a stabilisation of total forest loss rates during our second study period (2008–2014, Figure S6), i.e. there is no clear trend between 2008–2014 in deforestation rates.

We also compared GFC-based deforestation estimates for Brazil with estimates from PRODES. For direct comparison with PRODES, we used the annual primary forest mask provided by PRODES to re-calculate forest loss with the GFC product. This removes any inconsistency between products due to potential deforestation of non-forest areas and secondary forests, while still preserving differences due to different size thresholds considered. Comparison of the GFC forest loss temporal patterns for the Brazilian Amazon with deforestation data from PRODES revealed intriguing temporal differences between products. During the first three years of the study period (2001–2003), forest loss/deforestation estimates from GFC were on average ~34% lower than those of PRODES.

Over time, however, GFC forest loss estimates become progressively greater than PRODES deforestation estimates, so that over the last three years of the study period (2012–2014), GFC estimates of forest loss are ~72% greater than deforestation rates from PRODES (Figure S7), with maximum divergence observed in 2012 (the year in which the new Brazilian Forestry Code was enacted), when GFC forest loss estimates were greater than PRODES deforestation rates by a factor of 2.52. We find that this divergence can be at least partially explained by the increase in small-scale forest loss that are not incorporated within PRODES estimates. The fraction of total deforestation accounted for by patches <6.25 ha within the PRODES mask area increased from ~23% in 2004 to ~53% in 2013 (Figure S8).

## Discussion

### Emerging hotspots in Bolivia and Peru

Our findings paint a more complex picture of deforestation dynamics in Amazonia than has been reported thus far. While the widely-reported recent reduction in deforestation in the Brazilian Amazon is clearly observed in our analysis, we also highlight the growth of emerging deforestation hotspots in Bolivia and Peru. In 2001–2007, 99.5% of the area statistically defined as an Amazonian forest loss hotspot was found in Brazil, while in 2008–2014 this had fallen to 64%. This is associated with both the decline of deforestation in Brazil and the increasing importance of non-Brazilian deforestation hotspots. The declines in deforestation in the Brazilian Amazon are well-discussed in the literature and are likely associated with the implementation of the PPCDAm programme established in 2004^25^.

The new Peruvian hotspot, primarily in the Ucayali and San Martin regions of Peru appears to be linked to the growth of palm oil agrobusiness^26^. In the Ucayali region, palm oil production commenced in 2000 and steadily increased until 2010, with San Martin expanding between 2006 and 2012. The completion of the Pacific or Interoceanic Highway from Brazil to Matarani Port in Peru in 2010, may have acted as a vector for the incursion of people and subsequent forest loss in this region.

Similarly, large agricultural expansion also contributes as a driver of the deforestation hotspot in Santa Cruz, Bolivia. This region has been the focal centre of Bolivian deforestation for over two decades^27^, but our analysis shows a pattern of intensifying deforestation activity during the 2008-2014 period (Fig. 1), so that the Santa Cruz region now represents the largest hotspot of deforestation in Amazonia. Much of this deforestation appears to be linked to the expansion of the soybean sector, and may be associated with a leakage of soybean plantations from Brazil as a result of the soybean moratorium established in Brazil in 2006^28–30^.

### Changing size distribution of deforested patches

**Figure 5:**
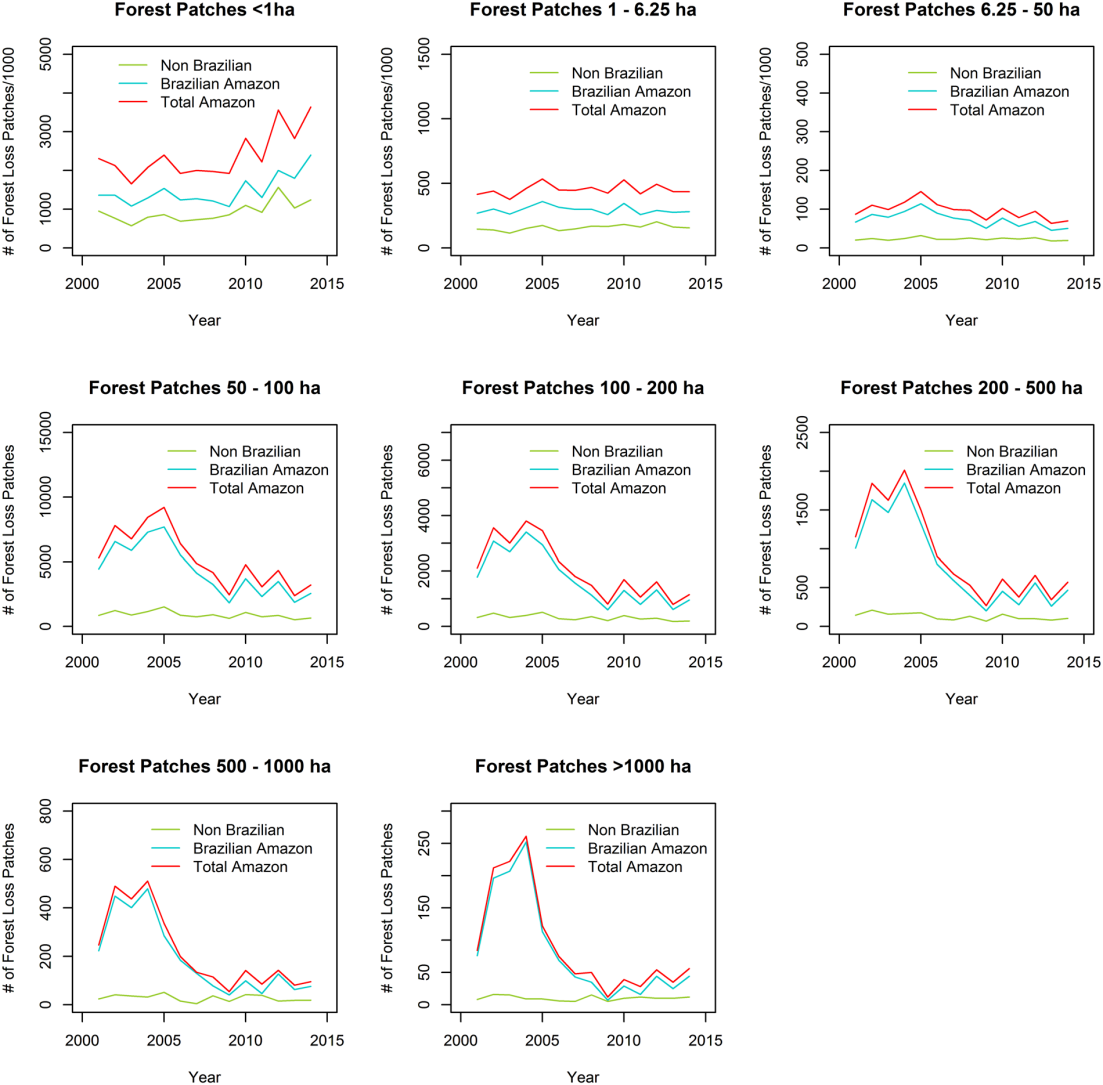
Forest loss across Brazilian and Non-Brazilian Amazon based on GFC Hansen *et al*. product according to patch sizes.

Our analysis also points to a considerable expansion of small-scale deforestation events between our two study periods (Fig. 2) which somewhat offsets the previously reported declines in larger deforestation patches^11^ based on analysis of the PRODES data. This pattern is widespread, with an increase in small-scale deforestation observed in almost all Amazonian countries (Fig. S2). Interestingly, the nature of these increases differs somewhat across countries. In Brazil and Bolivia, there is no obvious increasing trend up to 2008, after which there are progressive increases in small-scale deforestation. In the northern Amazonian countries (e.g. Guyana, French Guiana, Suriname), the temporal pattern is characterised by a sharp peak in 2012 (Fig. 5). Overall, our results lend support to recent findings suggesting an increased contribution of small landholders to deforestation in Brazilian Amazonia^31^, but we did not explicitly test this link between small patch size and small landholders. However, the different national patterns may be indicative of different deforestation drivers across regions.

The increasingly small size of deforestation patches in Brazil may also partially reflect attempts by larger landowners to evade monitoring of deforestation activities, which has recently been strengthened through the introduction of the PPCDAm program^32^. PRODES has a size cut-off of 6.25 ha, though deforestation events are collected at ~0.1 ha but not presented to maintain consistency with long-term data. Therefore, small-scale deforestation activities are only reported if they accumulate beyond that threshold. Additionally, PRODES may consider these small clearings as selective logging or ‘forests under use’ and not necessarily forest loss^33^. Some of the forest loss events reported in Hansen, *et al*.^22^ may also be due to the intense and increased degradation such as forest fires counted as deforestation events in the Hansen *et al*. product. Because of methodological differences between PRODES and GFC, which are based on contextual and pixel-based classifications, respectively, degradation processes will not appear in PRODES estimates, but may inflate small deforestation estimates based on GFC product. Therefore, causal attribution of small deforestation must be interpreted with caution. However, we should note that the same pattern is observed in Bolivia, where monitoring of deforestation and enforcement of penalties are not as effective as in Brazil^15^.

The 2012 peak in deforestation in Northern Amazonia coincides with the peak of the Amazon gold boom, marking the point when gold prices reached their highest historical value ($1,660/oz)^34^. Between 2012 and 2014, the price of gold fell by 28%; during this time, small forest loss activity in French Guiana, Guyana and Suriname declined back to baseline rates. Gold mining has recently been highlighted as an important driver of deforestation in both Peru^21^ and northern Amazonia more generally^35^. Our analysis supports these findings but also suggests that, in the Guianas at least, the increases in deforestation associated with gold mining were relatively short-lived.

### Geographic spread of small-scale forest loss

Small-scale forest loss events have not only increased in number but have also increased greatly in geographical spread across Amazonia. Forest loss density (in km^2^ per 100 km^2^) increased by an order of magnitude or more across large areas of the Amazon typically associated with very low deforestation rates between 2001–2007 and 2008–2014 (Fig. 3). This phenomenon occurs across the Amazon Basin, highlighting the dispersed nature of small forest loss events. Thus, remote areas of the Amazon, largely thought to be isolated from deforestation pressures^36^, are now being impacted much more than before.

The small-scale nature of these events means that ultimately they would not be reported by monitoring systems such as PRODES which assume a much higher size threshold to be computed as deforestation. Additionally, in response to increasing awareness of rising small-scale forest loss events, the Brazilian government had plans to update its near real-time deforestation detection system (DETER)^37^, which uses lower spatial resolution images than PRODES but with high temporal frequency, so that the threshold for deforestation has been reduced to 3 ha. Despite the DETER data being at a lower threshold, and in real-time, it is only available on a 3-monthly basis and not included in the national deforestation statistics. This current trend of increasing small clearings would indicate that conservation efforts may now need to focus on fighting degradation and low density deforestation as the large deforestation events are now relatively under control, despite an increase in these large scale events in 2015 and 2016 (Marcos Adami, personal communication). Our study suggests that inclusion of small deforestation patches in national estimates is very important, given the growing divergence we found between PRODES and GFC estimates (Fig. S8).

Attribution of the specific causes of observed increases in small-scale forest loss is challenging. However, there are very strong reasons to believe that the increased small-scale forest loss events observed are linked to anthropogenic activity rather than natural disturbances. Most natural disturbance events in Amazonia occur at the sub-pixel scale. For example, Espirito-Santo, *et al*.^38^ found that 98.6% of natural carbon losses in Amazonia are due to small-scale mortality events (<0.1 ha) with mean disturbance areas of 0.01 to 0.03 ha across different field-based and LiDAR datasets, equivalent to 1/9 to 1/3 of a Landsat pixel. This of course does not exclude the possibility that there has been an increase in larger natural disturbance events between both periods. We find no clear evidence, however, of anomalous patterns in small-scale forest loss in 2005 and 2010, the two large drought years encompassed by our study (Figure S5).

Additional analysis also suggests that the observed increase in small deforestation events was not overly influenced by artefacts introduced by processing and classification problems. First, we found that our results were robust to the version of the GFC product used (Fig. S9 and S10), over the period for which we had data for both versions (2001-2012). Second, we found that the relative differences in increases in small-scale deforestation intensity between our two focal periods was robust to changes in the specific lower bounds used to delimit the ‘light’ deforestation category, with relative patterns between periods being maintained even after doubling and trebling of assumed deforestation cut-offs for that class (SI Figure 11). Although Hansen, *et al*.^22^ report a global accuracy of 99.5% for tropical regions, there are still some misclassification errors, as there are with all large-scale deforestation products. However, we can think of no major reasons beyond changes in processing, evaluated by comparing two versions of the GFC product, to expect classification accuracy to vary over time.

In many areas, especially in the northern Amazonian countries (French Guiana, Guyana, Suriname, Venezuela), the geographical spread of small-scale deforestation events expanded greatly in 2012 (Figure S3), mirroring the large increase in small deforestation events seen that year associated with the Amazonian gold boom. This suggests that the spatial footprint of deforestation tied to gold-mining activities in those countries is much greater than previously reported^35^, implying an important role of such activities in opening up remote areas of Amazonia to deforestation pressure, even if for short-lived periods.

### Implications for conservation efforts

Our results also suggest that the protected area networks in Amazonia have had limited success combating the pervasive spread of small-scale deforestation, as the relative increases in small-patch deforestation events were similar inside and outside protected areas over the two study periods. These small-scale losses in forest cover now present a new and alarming challenge for conservation efforts in Amazonia, as they are inherently more difficult to monitor and control. Protected areas are seen as a cornerstone for reducing deforestation and carbon emissions. Our results suggest that the management strategies of Amazonian protected areas may need revising to account for the increasing threat of low-density, small-scale forest losses.

In summary, our analysis of the high-resolution GFC data for Amazonia revealed three important insights. First, centres of high intensity deforestation have shifted away from the traditional arc of deforestation to Bolivia, Peru and the northeastern Brazilian Amazon. Second, there has been a marked increase in small-scale deforestation, partially offsetting the previously reported declines in deforestation of larger patch sizes. Thirdly, light deforestation events have spread pervasively across the entire Amazon in recent years, even in protected areas. Altogether these results raise awareness of new threats that national-level statistics do not capture and pose new challenges for conservation of Amazonian forests.

## Methods

### Deforestation datasets and overarching temporal patterns

We defined our Amazonian area of interest following the boundaries proposed by Eva and Huber^39^. This includes sections of nine countries: French Guiana, Suriname, Guyana, Venezuela, Colombia, Ecuador, Peru, Bolivia and Brazil. We obtained deforestation data for our area of interest from the 30-m resolution Global Forest Change (GFC) data of Hansen, *et al*.^22^, based on Landsat imagery. The Hansen, *et al*.^22^ data was downloaded from http://earthenginepartners.appspot.com/science-2013-global-forest (Version 1.2). All results shown are for GFC Version 1.2, as this allowed analysis of the longest time series available. However, we also compared our results with GFC Version 1.0 data over the period for which we had data for both products (2001–2012). This was done to explore the potential effects of reprocessing changes in v. 1.2, where the reprocessing algorithm was changed for the 2011–2014 period. In v 1.0, consistent processing was used throughout the entire time series (2001–2012). We applied no further processing to the Hansen *et al*.^22^ dataset. Our analysis indicated that overall patterns were very similar for both versions of the GFC data, over the period where both versions were available (SI Figures S9 and S10). Our study region spanned thirteen GFC (Landsat) tiles which were clipped to match the boundaries of our area of interest. We restricted our analysis to the Tropical Broadleaf Forest biome using the definition and extent from Nature Conservancy (http://maps.tnc.org/gis_data.html). We defined as forest those pixels in GFC with >30% tree cover as per Kim, *et al*.^40^. The total area deforested in the Amazonian region of each country was calculated annually for the GFC dataset (2001–2014). In the GFC dataset, deforestation was defined as a stand-replacement disturbance or the complete removal of tree cover canopy at the Landsat pixel scale^22^; with the exclusion of pixels after a deforestation event annually. Forest gain areas were therefore not considered in our analysis. The PRODES shapefile was derived from http://maps.csr.ufmg.br/, and indicates annual deforestation rates in the Brazilian Amazon since 1997. To compare PRODES data with the GFC product, we created yearly masks using the PRODES primary forest and associated yearly forest loss pixels to subset annual GFC data. This allowed the removal of all non-primary forests and non-forest regions from the analysis. Maps in Figs 1,3,4, S1, S5, S9 and S11 were generated using ArcGIS 10.4.1 software by Esri (Environmental Systems Resource Institute, www.esri.com).

For all analyses described below we divided our dataset into two periods of equivalent length: 1) 2001–2007 and 2) 2008–2014. We statistically evaluated the differences in means of relevant metrics for both periods using the non-parametric Wilcoxon’s signed rank test as the data was not normally distributed.

### Analysis of size dynamics of deforested patches

We defined deforested patches as contiguous areas of forest that were cleared within a year. Deforested patches were classified into eight categories, namely ≤1 ha, ≤6.25 ha, 6.25–50 ha, 50–100 ha, 100–200 ha, 200–500 ha, 500–1000 ha and >1000 ha. This is the same size class breakdown used by Rosa, *et al*.^11^, with the exceptions that we also consider deforestation patches ≤1 ha and ≤6.25 ha, which are below the annual deforestation threshold detected by the PRODES data analysed in this study.

### Analysis of spatial patterns of deforestation

The GFC data were also used to investigate spatial patterns of deforestation density (number of deforested pixels per area), including their temporal dynamics using ArcGIS 10.4.1. To visualise deforestation density, we created a 10 × 10 km grid over our study area, within which we classified total deforested area within each gridcell into five categories following a log scale: (1) Negligible (<0.01 km^2^ per 100 km^2^ land area), (2) Light (0.01–0.1 km^2^ per 100 km^2^ land area), (3) Moderate (0.1–1 km^2^ per 100 km^2^ land area), (4) Heavy (1–10 km^2^ per 100 km^2^ land area) and (5) Very Heavy (>10 km^2^ per 100 km^2^ land area). Annual data were grouped into two periods for analysis: 2001–2007 and 2008–2014. We conducted additional sensitivity analyses to explore the implications of the assumed lower boundaries for ‘light’ deforestation on our results. To do this, we performed additional calculations where the lower boundary was doubled (set to 0.02 km^2^ per 100 km^2^) or trebled (set to 0.03 km^2^ per 100 km^2^). To evaluate whether similar patterns were observed inside and outside of protected areas, we applied a protected area mask using shapefiles provided by The World Database on Protected Areas (WDPA), which include protected areas from all IUCN categories as well as indigenous protected areas, biological and biosphere reserves, cultural sites, sustainable reserves and hunting preserves (www.protectedplanet.net). We conducted separate Wilcoxon signed rank tests for areas inside and outside of protected areas to investigate changes in deforestation density between our two focal periods (2001–2007 and 2008–2014).

For each of our two study periods (2001–2007 and 2008–2014), we also statistically evaluated the spatial clustering patterns of deforestation, using the Getis-Ord Gi* statistic^41^ in ArcGIS 10.4.1. Getis-Ord Gi* is a local clustering statistic and was used to statistically determine the occurrence of deforestation hotspots^41^. The statistic is ${G}_{i}^{\ast }(d)=\frac{\sum _{j=1}^{n}{w}_{ij}(d)\cdot {x}_{j}}{\sum _{j=1}^{n}{x}_{j}}$ where *x*_j_ is the number of spatially mapped deforested 30-m pixels within a 10 × 10 km gridcell *j*; *w*_ij_ (*d*) is a weights matrix with values equal to 1 for gridcells located within a distance *d* from gridcell *j* and zero otherwise, and n is the total number of mapped deforestation rates (at locations j = 1, … n). These analyses were conducted using the spatial statistics tools in ArcGis 10.4, using a fixed distance *d* of 100 km, where features within this distance are its neighbours while features further away are not. The distance of 100 km was selected after running multiple Moran’s I statistics for a peak in z-scores. Hotspots (clustered deforestation patches) have comparably large positive Gi* values while absence of clustering is indicated by Gi* values close to zero. To detect hotspots Z-scores (standard deviations of all Gi* values) and corresponding p-values for Gi* were calculated for each gridcell to determine whether forest loss in a given gridcell is statistically clustered relative to neighbouring gridcells and compared to all gridcells in our study region^41^. Significant positive Z-scores denote local clustering of high deforestation counts indicating a significant hotspot.

## Electronic supplementary material

Supplementary Information
