# Supplementary material for: Pervasive Rise of Small-scale Deforestation in Amazonia
Source: Sci Rep. 2018 Jan 25;8:1600. doi: 10.1038/s41598-018-19358-2 (PMC5785515; doi:10.1038/s41598-018-19358-2)
Supplement: Supplementary file 1 — Supplementary Information [file 41598_2018_19358_MOESM1_ESM.pdf]

Pervasive Rise of Small-scale Deforestation in Amazonia

Michelle Kalamandeen[1*], *Emanuel Gloor*[1], *Edward Mitchard*[2], *Duncan Quincey*[1], *Guy Ziv*[1], *Dominick Spracklen*[3], *Benedict Spracklen*[3], *Marcos Adami*[4], *Luiz E. O.C. Aragão*[5,6], *David Galbraith*[1*]

Author Affiliations

1. School of Geography, University of Leeds, Leeds, UK, LS2 9 JT.
2. School of GeoSciences, University of Edinburgh, Edinburgh, UK, EH9 3FF.
3. School of Earth and Environment, University of Leeds, Leeds, UK, LS2 9JT.
4. Instituto Nacional de Pesquisas Espaciais (INPE), Belém, Pará, Brazil, CEP: 66077-830.
5. Instituto Nacional de Pesquisas Espaciais (INPE), Sao José dos Campos, São Paulo, Brazil, CEP: 1227-010.
6. College of Life and Environmental Sciences, University of Exeter, EX4 4RJ, UK

SUPPLEMENTAL INFORMATION

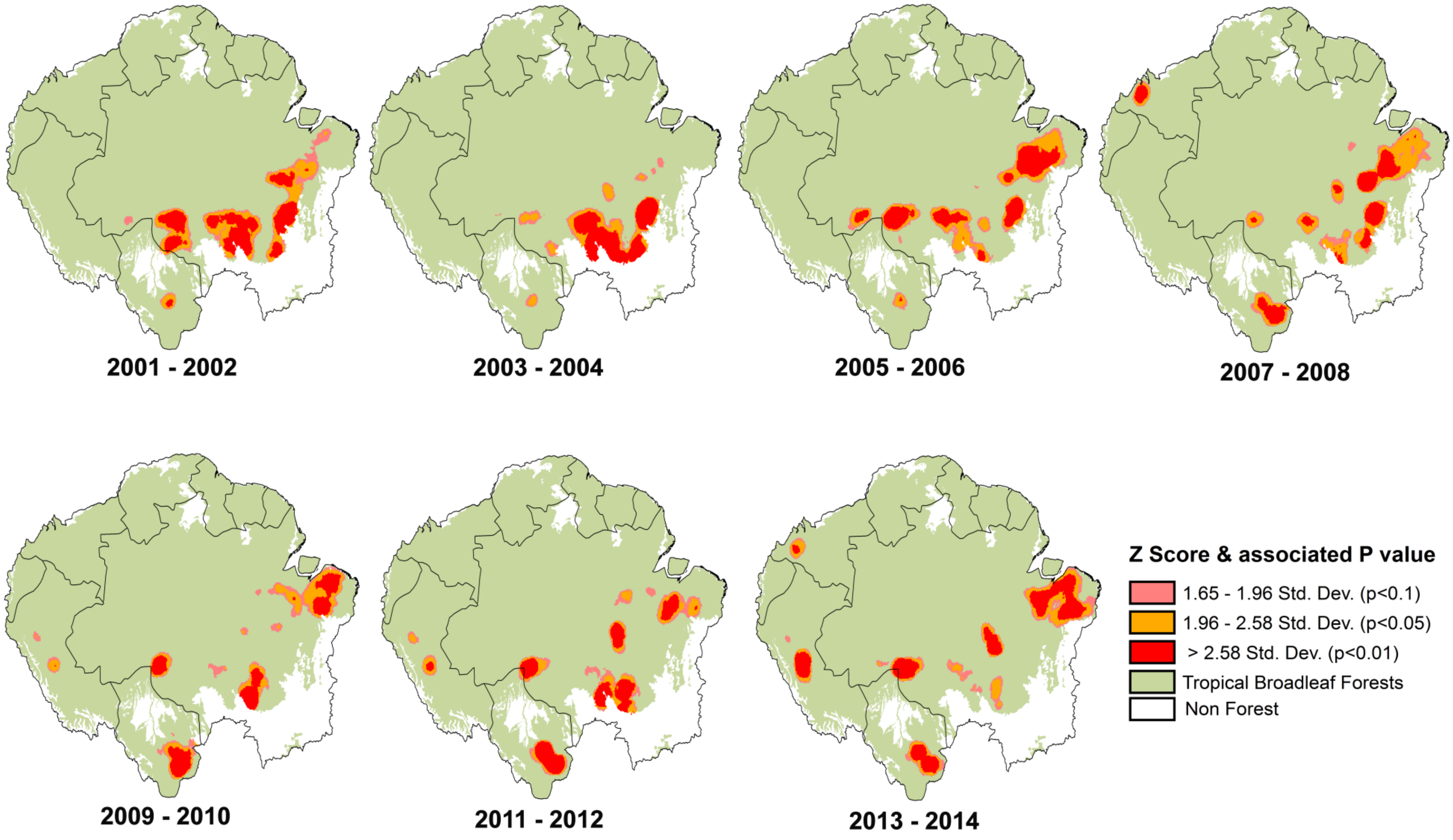

Figure S1: Bi-annual hotspots of forest loss (km^2) across Amazonia (2001-2014) using the Getis-Ord Gi* analysis in ArcGIS 10.4.1 (www.esri.com).

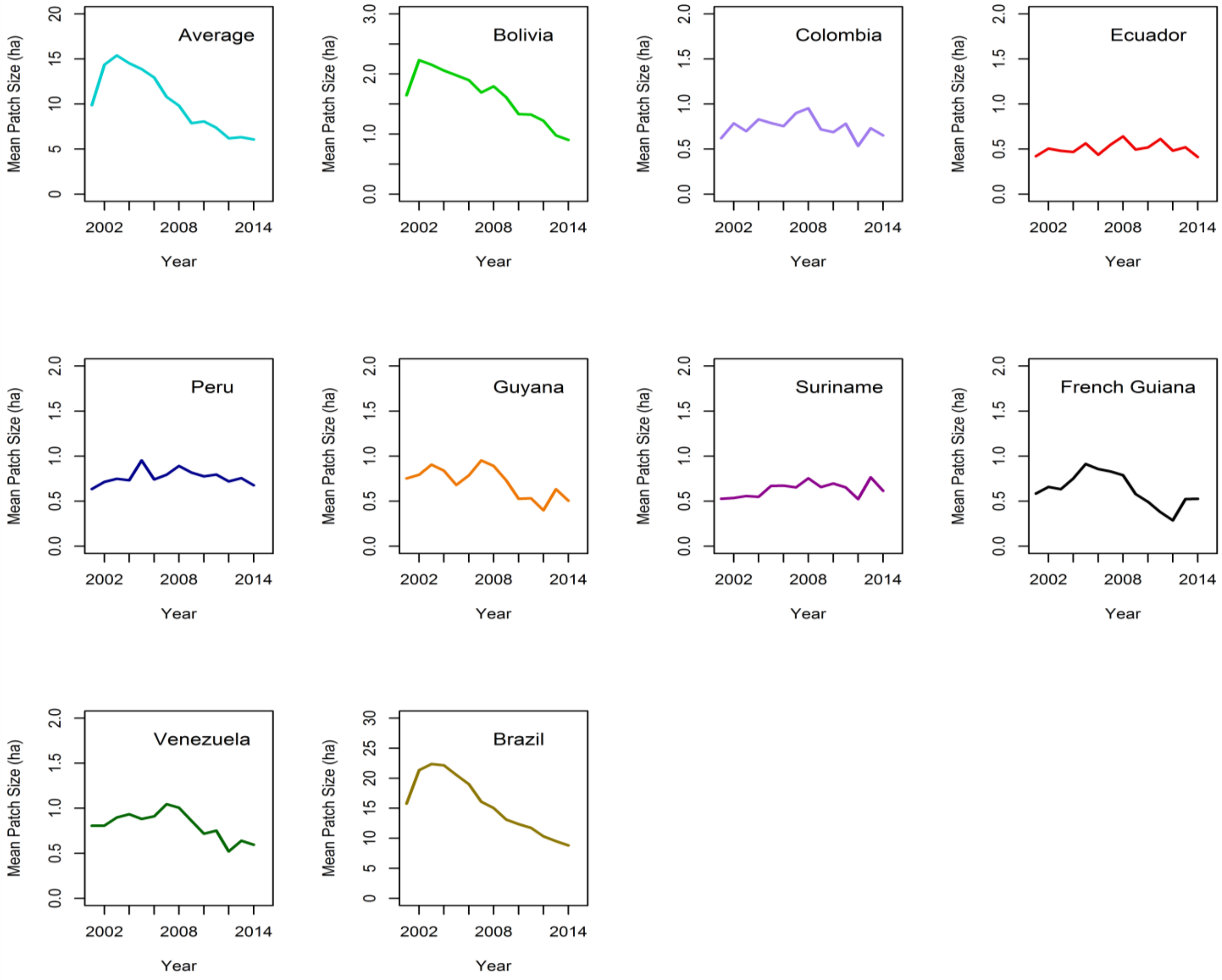

Figure S2: Mean Deforested Patch Sizes (ha) across Amazonia, 2001-2014.

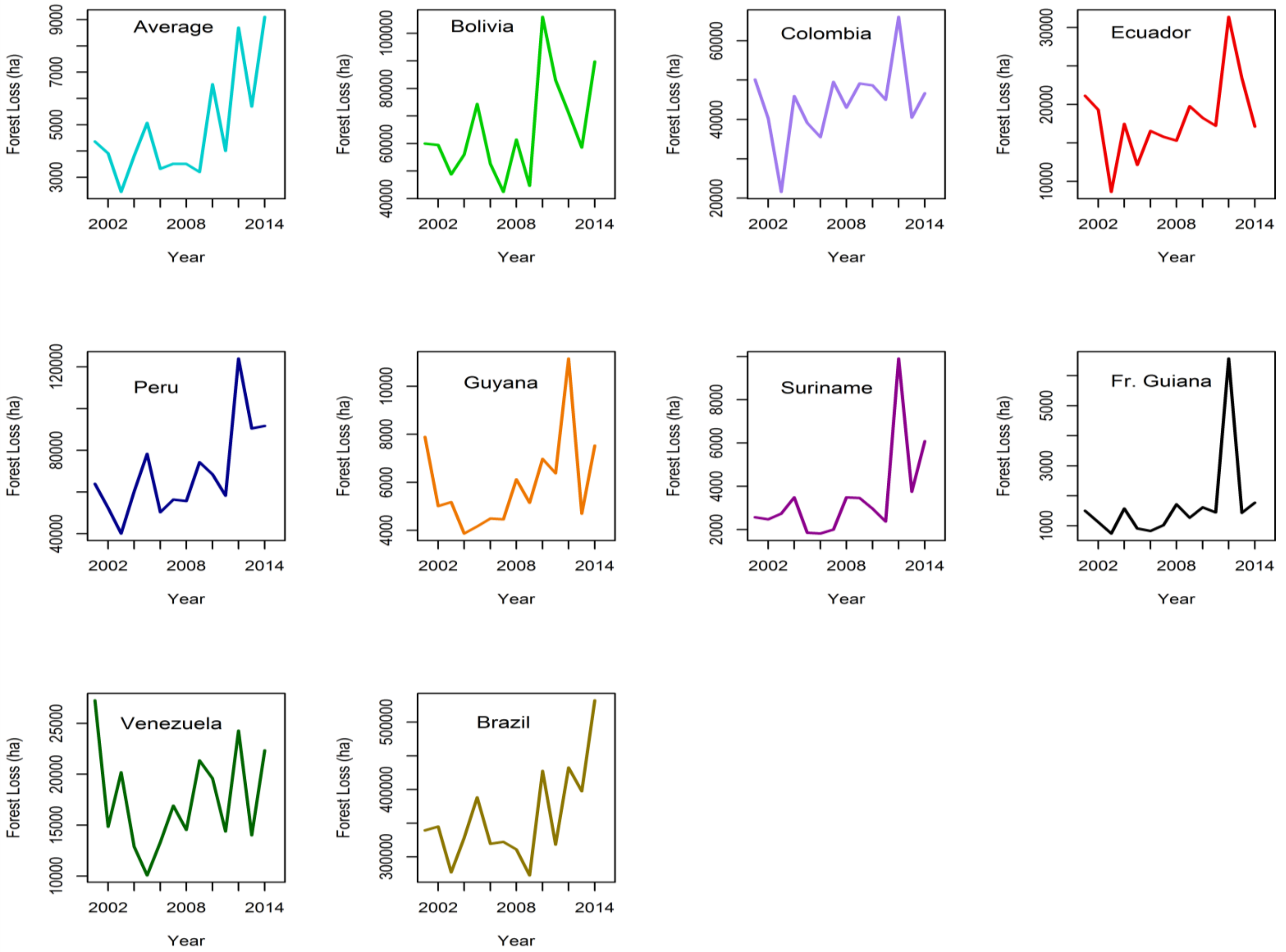

Figure S3: Trajectory of area of small-scale forest loss (<1 ha) across Amazonia, 2001-2014.

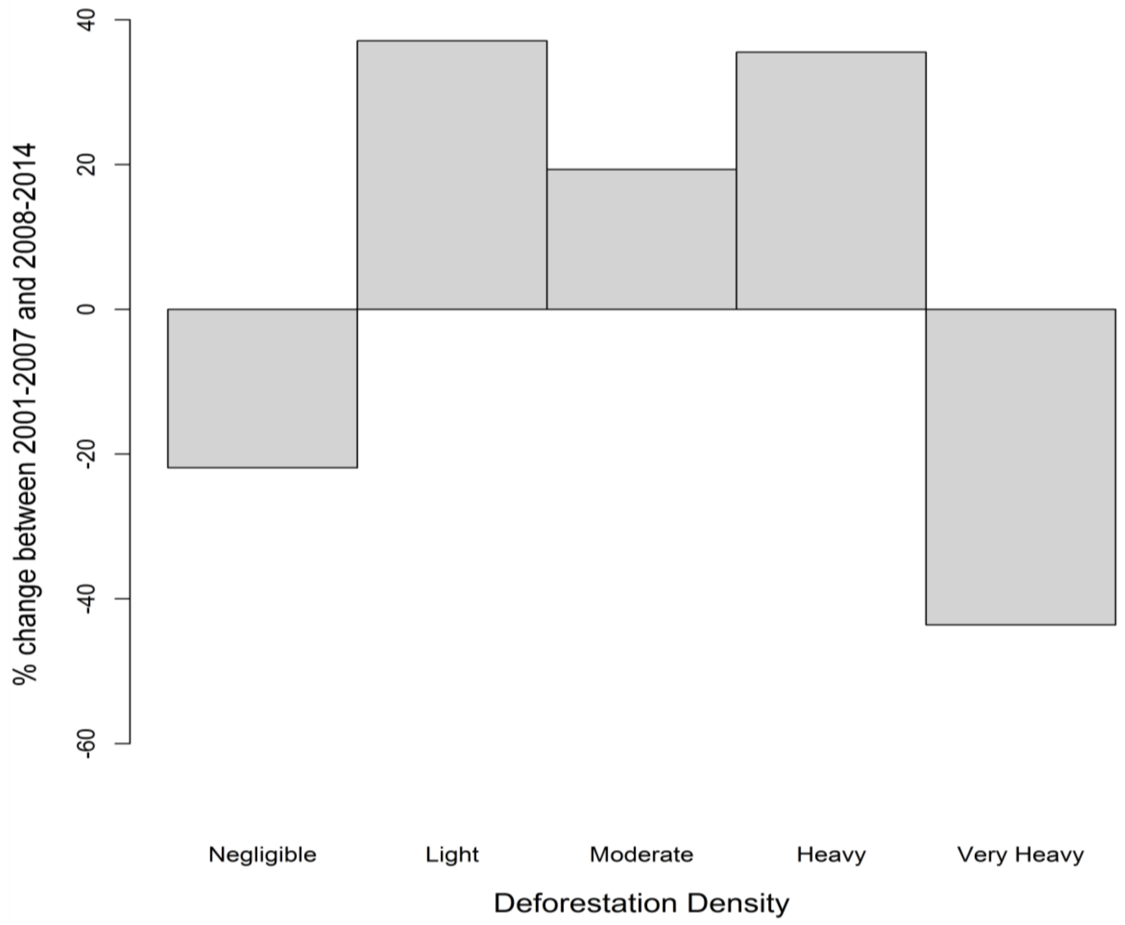

Figure S4: Change (%) in number of 10 x 10 km gridcells between 2001-2007 and 2008-2014 for five different forest loss density categories. Negligible: < 0.01 km^2 / 100 km^2. Light: 0.01 – 0.1 km^2 /100 km^2. Moderate: 0.1 – 1 km^2/ 100 km^2. Heavy: 1-10 km^2/100 km^2. Very heavy: 10-100 km^2/100 km^2.

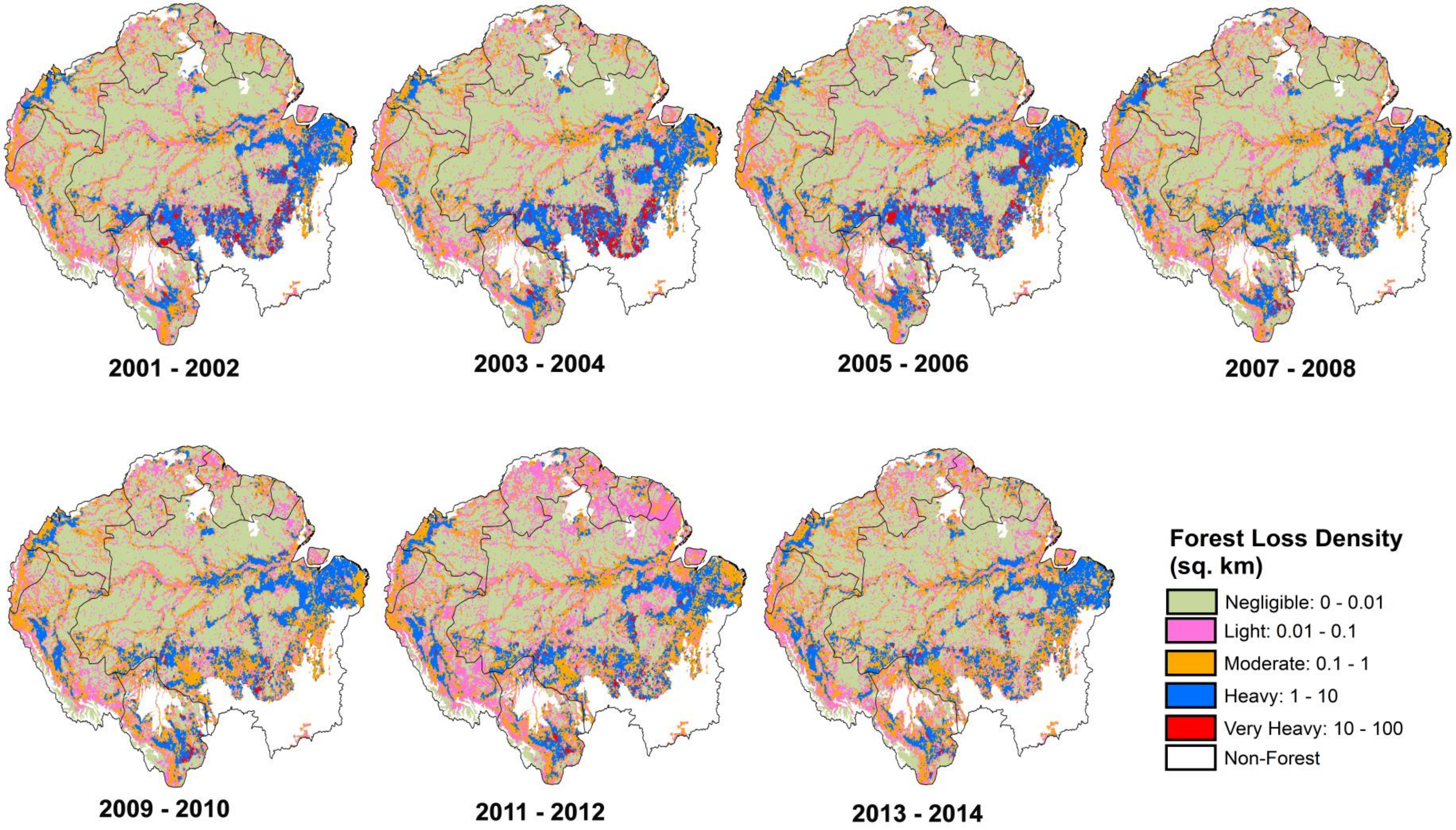

Figure S5: Bi-annual forest loss density (km^2 deforested area over the target period per 100 km^2 land area) across Amazonia (2001-2014) using ArcGIS 10.4.1 (www.esri.com).

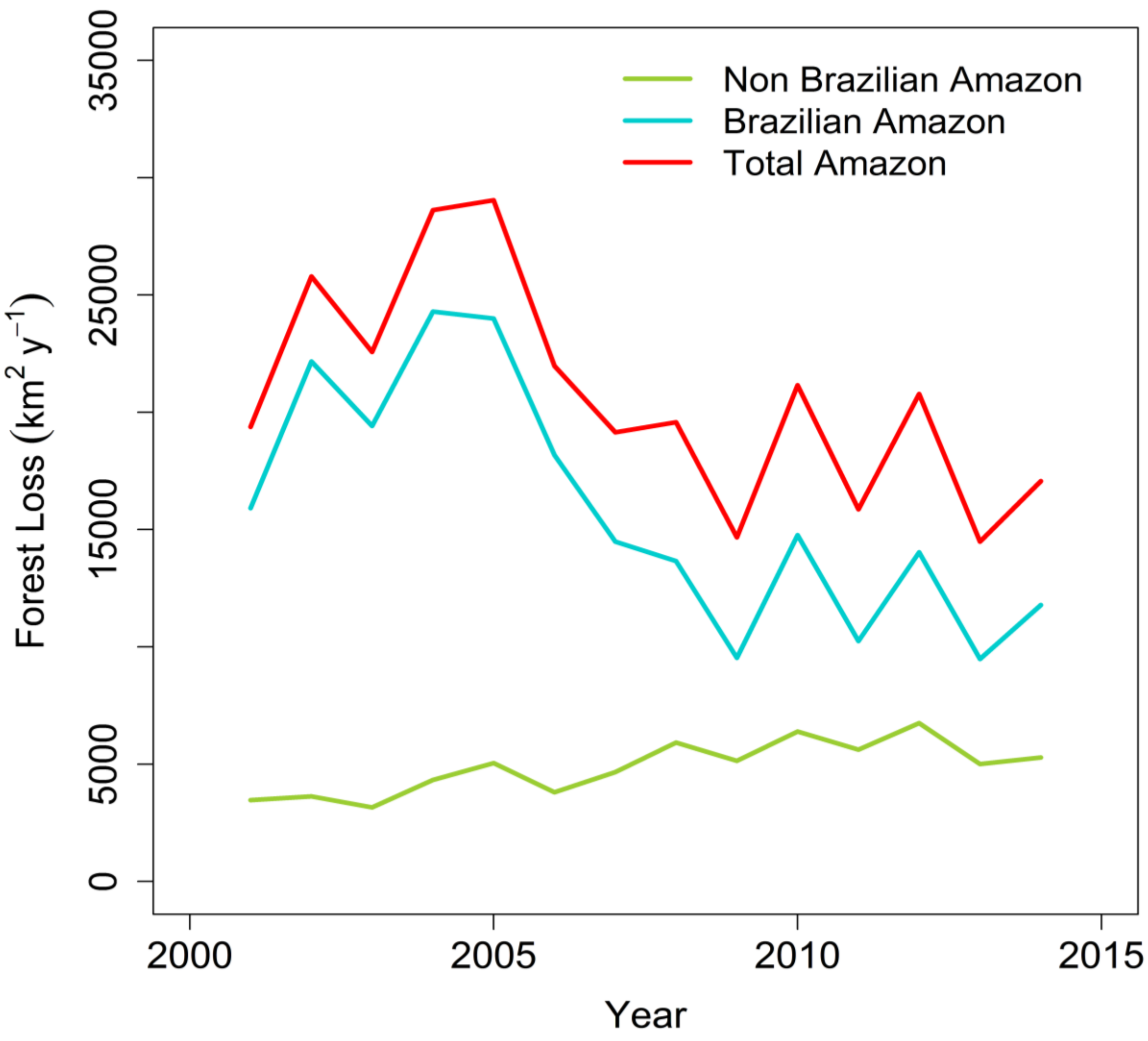

Figure S6: Annual forest lost (km^2) across Amazonia for 2001-2014, based on the Global Forest Change (GFC) product.

Country	GFC		
	Annual mean loss rate		Significance of mean
	2001-2007	2008-2014	
**Bolivia**	79.39	175.62	0.04**
**Colombia**	65.29	-24.47	0.7
**Ecuador**	-1.22	0.71	0.01**
**Peru**	70.84	130.85	0.004**
**French Guiana**	0.11	0.26	0.62
**Guyana**	-4.87	-0.76	0.02**
**Suriname**	0.32	11.62	0.001***
**Venezuela**	-1.94	-8.09	0.1
**Brazil**	-273.66	-230.22	0.001***

***P < 0.1**
**** P < 0.05**
***** P < 0.001**

Table S1: Significance (*p*-value) of forest loss (km^2) across Amazonia for *Hansen et al* GFC product, 2001-2014. The significance was calculated on the means of the two time periods (2001-2007 & 2008-2014) using the Wilcoxon signed-ranked test.

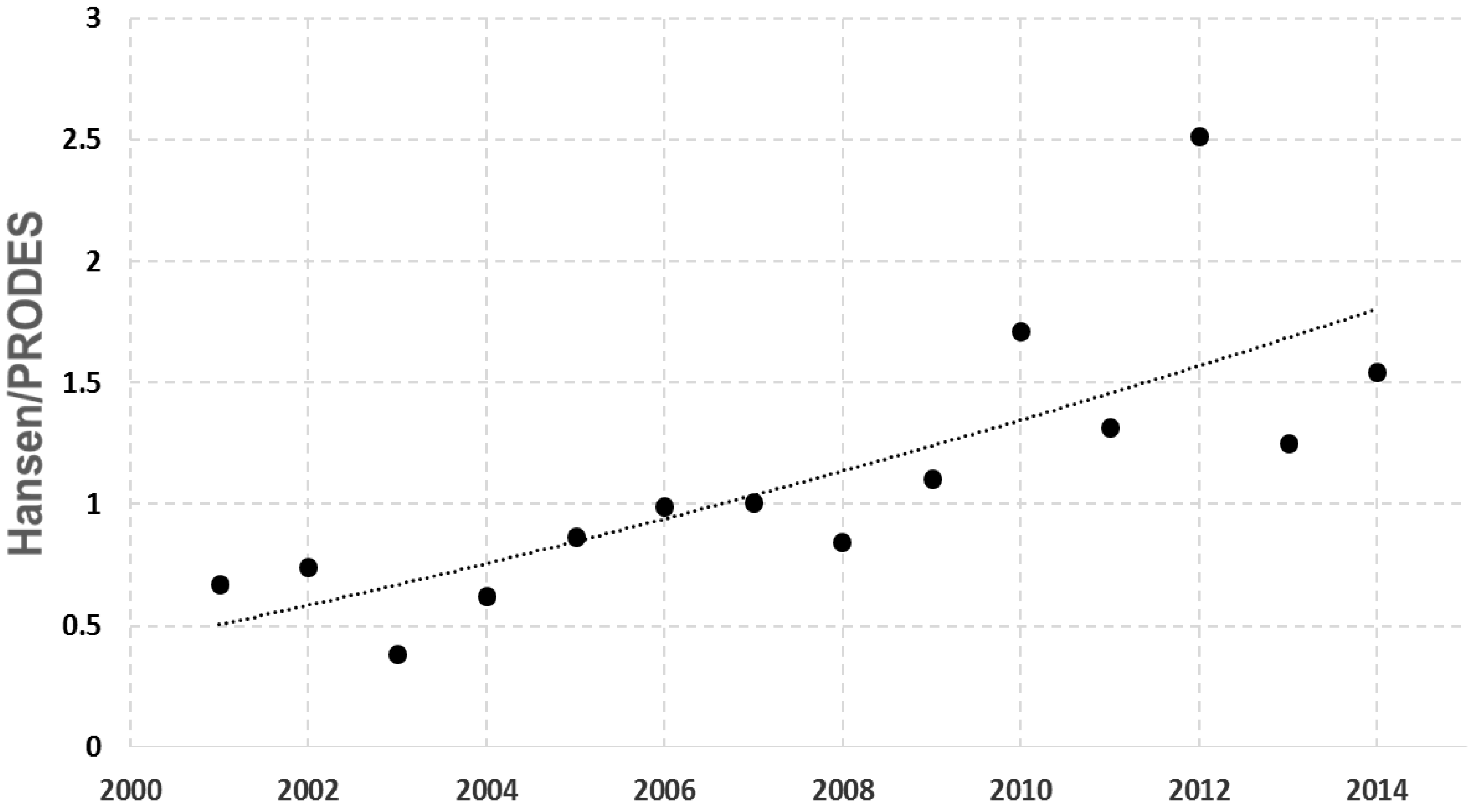

Figure S7: Ratio of annual forest loss area for the Brazilian Amazon estimated from Hansen *et al*. GFC product relative to PRODES deforestation estimates.

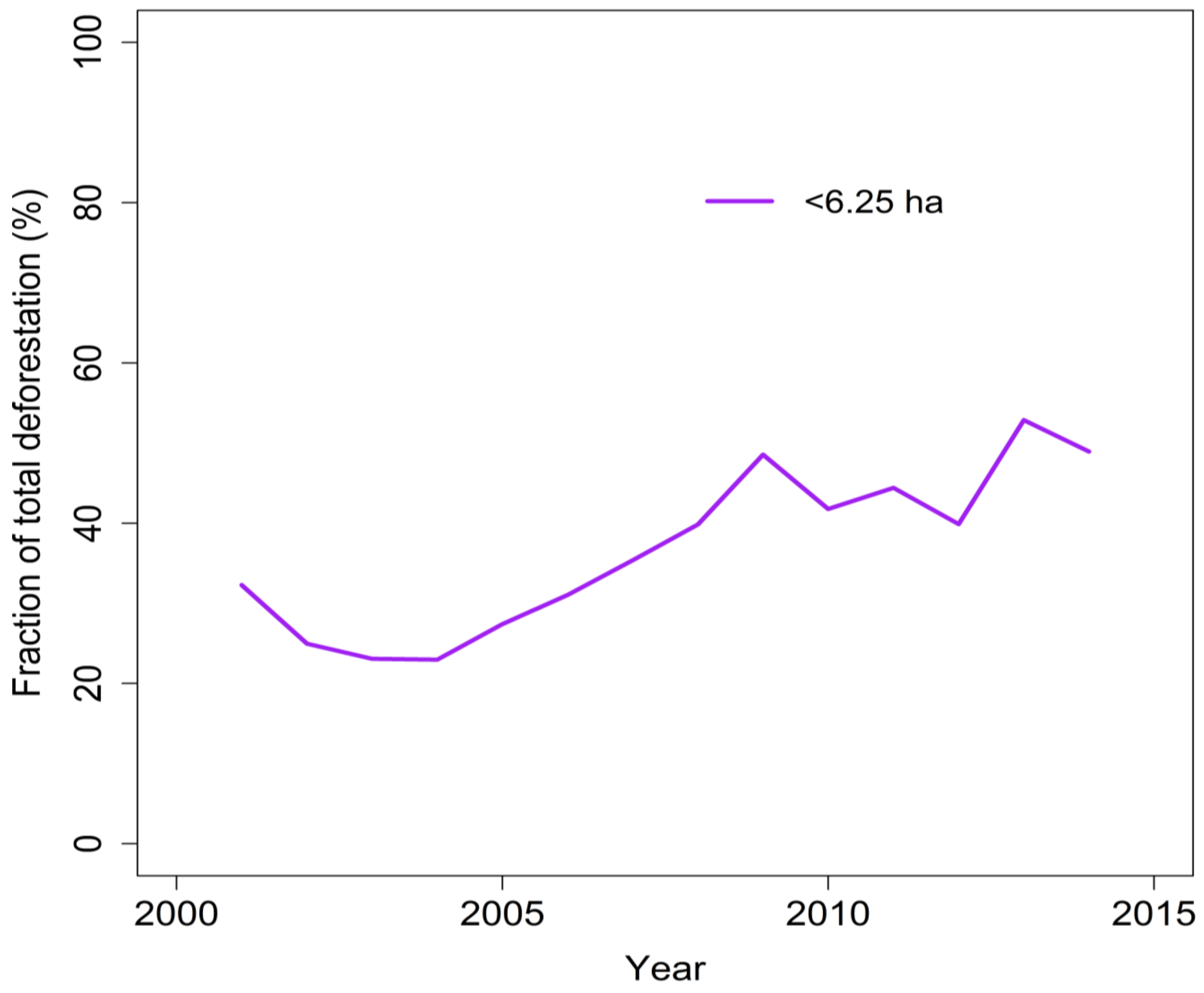

Figure S8: The fraction of total Hansen *et al*. GFC deforestation attributed to small patches <6.25ha (PRODES threshold).

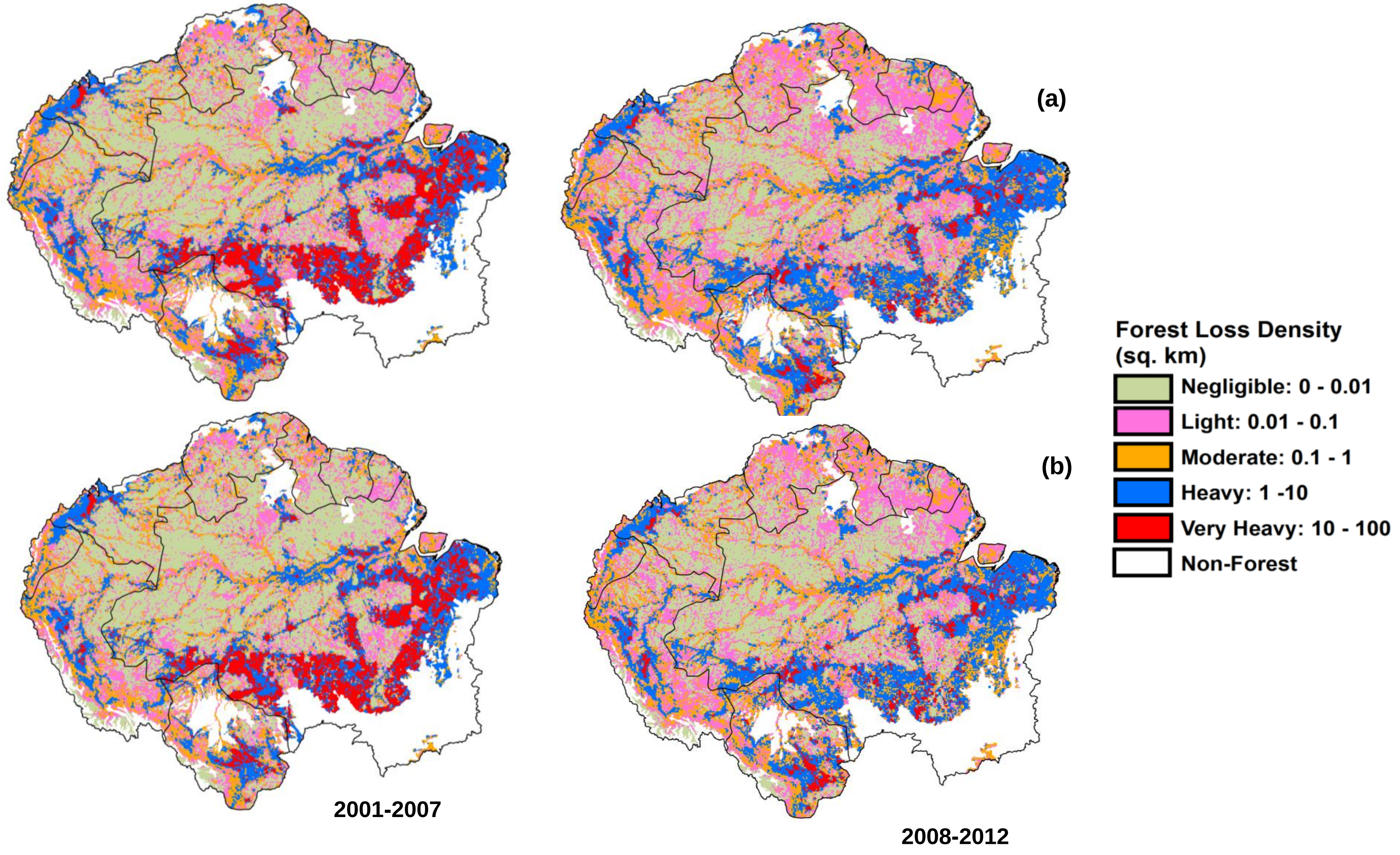

Figure S9: Comparison of forest loss density (km^2 forest loss per 100 km^2 land area) in Amazonia using the GFC (a) version 1.0 and (b) version 1.2 products for two time periods: 2001-2007 and 2008-2012 using ArcGIS 10.4.1 (www.esri.com).

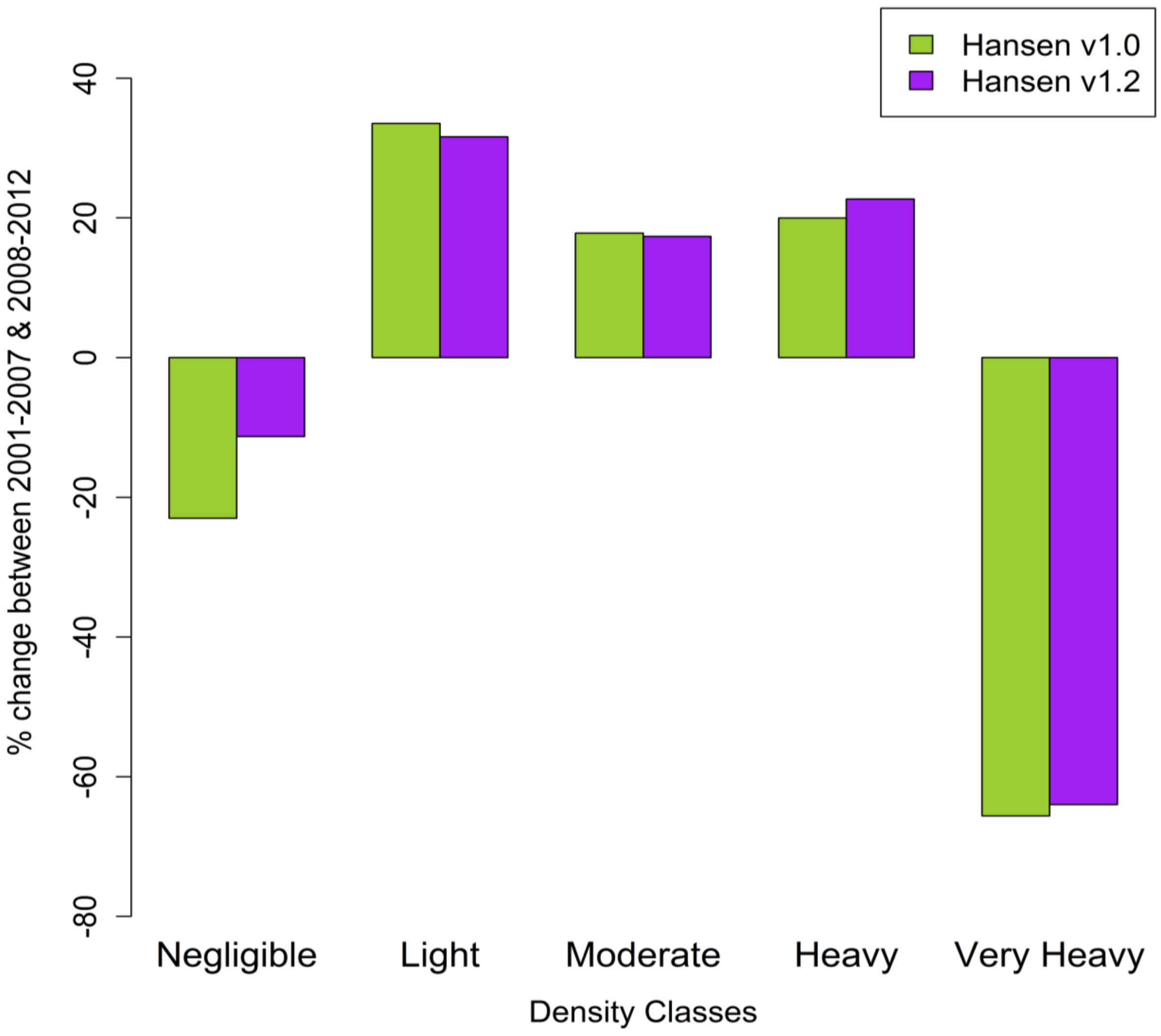

Figure S10: Percent change of forest loss density categories for GFC versions 1.0 and 1.2 for 2001-2007 and 2008-2012 across Amazonia.

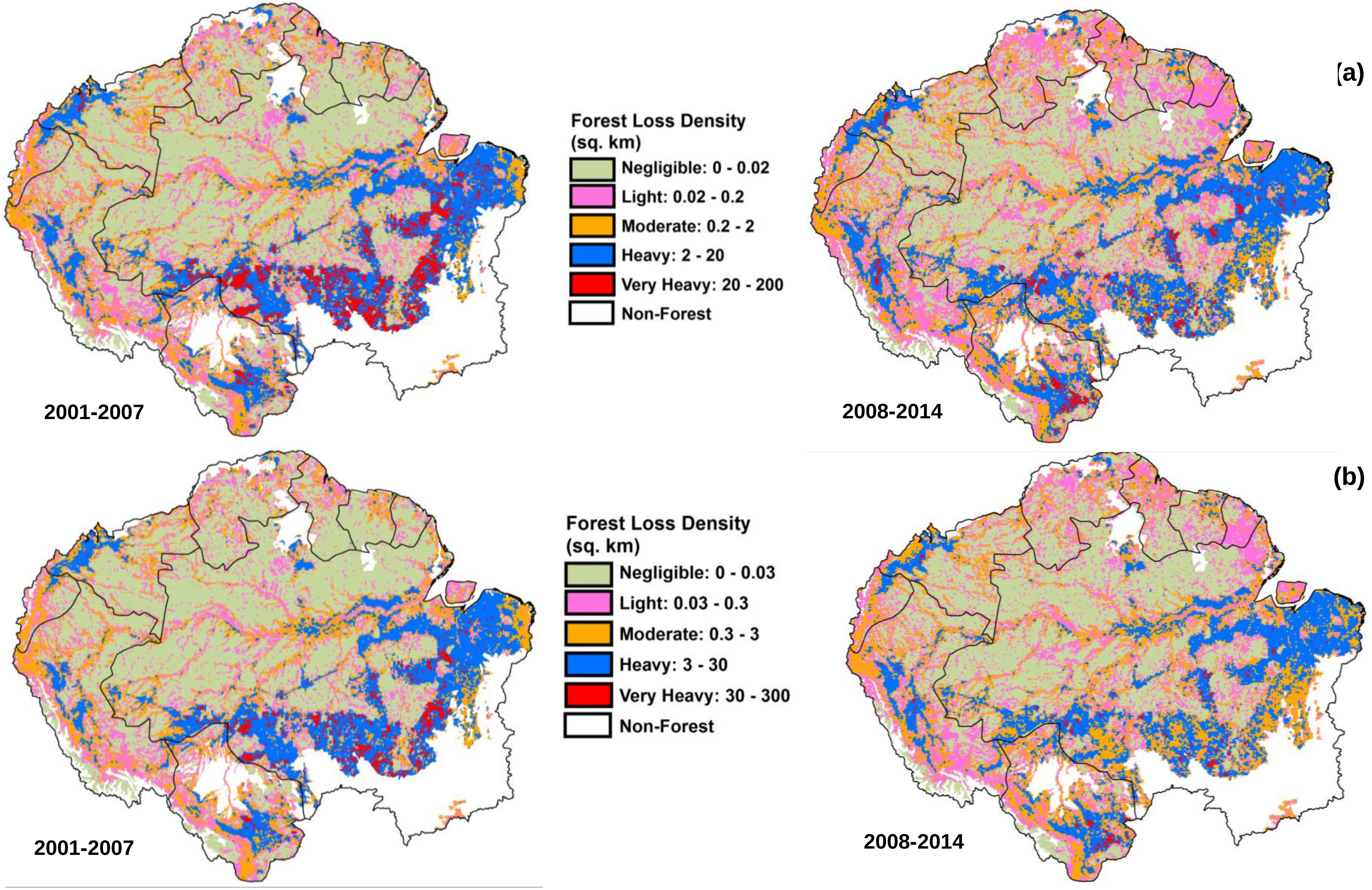

Figure S11: Forest loss density (km^2 forest loss per 100 km^2 land area) in Amazonia, as calculated using the GFC Version 1.2 product for two time periods: 2001-2007 and 2008-2014 using ArcGIS 10.4.1 (www.esri.com). Here, we (a) doubled and (b) trebled the lower classification to examine whether classification influenced our density results. Relative differences were sustained at 15.6% between 2001-2007 and 2008-2014 across all classifications.
